# The Protective Effect of Sulforaphane on Dextran Sulfate Sodium-Induced Colitis Depends on Gut Microbial and Nrf2-Related Mechanism

**DOI:** 10.3389/fnut.2022.893344

**Published:** 2022-06-27

**Authors:** Canxia He, Mingfei Gao, Xiaohong Zhang, Peng Lei, Haitao Yang, Yanping Qing, Lina Zhang

**Affiliations:** ^1^The Affiliated Hospital of Medical School, Ningbo University, Ningbo, China; ^2^Institute of Preventative Medicine, School of Medicine, Ningbo University, Ningbo, China; ^3^Center for Engineering in Medicine and Surgery, Department of Surgery, Massachusetts General Hospital, Boston, MA, United States; ^4^Department of Pathology, Mingzhou Hospital of Zhejiang University, Ningbo, China

**Keywords:** sulforaphane, ulcerative colitis, gut microbiota, Nrf2, inflammation, intestinal barrier, inflammatory bowel disease

## Abstract

Sulforaphane (SFN), an isothiocyanate present in cruciferous vegetables such as broccoli and brussels sprouts, has a variety of biological functions. This study was undertaken to assess the potential efficacy of SFN in ameliorating dextran sulfate sodium (DSS)-induced ulcerative colitis (UC) in mice and to elucidate the underlying mechanisms. UC was induced in mice with administration of 2% DSS in drinking water for 7 days. Male C57BL/6 mice were treated with Mesalazine (50 and 100 mg/kg body weight) and various doses of SFN (2.5, 5, 10, and 20 mg/kg body weight). In DSS colitis mice, the hallmarks of disease observed as shortened colon lengths, increased disease activity index (DAI) scores and pathological damage, higher proinflammatory cytokines and decreased expression of tight junction proteins, were alleviated by SFN treatment. SFN also partially restored the perturbed gut microbiota composition and increased production of volatile fatty acids (especially caproic acid) induced by DSS administration. The heatmap correlation analysis indicated that *Lactobacillus johnsonii, Bacteroides acidifaciens*, unclassified *Rikenellaceae RC9*, and unclassified *Bacteroides* were significantly correlated with disease severity. Nuclear factor (erythroid-derived 2)-like 2 (Nrf2), Signal Transducer and Activator of Transcription 3 (STAT3), and Phase II enzyme UDP-glucuronosyltransferase (UGT) were involved in the protective effect of SFN against DSS-induced colitis. This study's findings suggest that SFN may serve as a therapeutic agent protecting against UC.

## Introduction

Inflammatory bowel disease (IBD), including Crohn's disease and ulcerative colitis (UC), is a chronic inflammatory disease of the gastrointestinal tract. Over 1 million residents in the United States and 2.5 million across Europe are estimated to have IBD with substantial costs for health care (1). Even in China, owing to the industrialization of society, incidence and prevalence of IBD has strikingly increased (2). Diet, frequent antibiotics use, and smoking are potential risk factors in IBD (3). The complex and precise underlying mechanisms are yet to be completely understood, however, the intestinal barrier function, immune system, and cross-talk between host and gut microbiota, are various processes known to be involved in the pathogenesis of IBD (4).

The disruption of intestinal barrier function is a hallmark of IBD. This leads to increased intestinal permeability and a significant impact on the gut microbial composition and diversity. Emerging evidence highlights the pivotal role played by gut microbiota in maintaining normal physiological functions of colon along with regulation of local immune system. An altered intestinal barrier and immune signaling dysregulation lead to mucosal inflammation, associated with an increased secretion of pro-inflamatory cytokines (5). Pro-inflammatory cytokines like interleukin-6 (IL-6) and tumor necrosis factor TNF-α), have been demonstrated as substantially upregulated, and strongly correlate with the severity of active IBD. Moreover, elevated levels of these cytokines, such as TNF-α, are known to damage intestinal tight junctions and permeability (6). Subsequently, an ongoing cycle ensues between the intestinal immune system, gut microbiota, and barrier function, responsible for the various clinical presentations of IBD. Given the multi-factorial etiology and complex pathophysiology of IBD, existing therapies have fallen short in terms of limited efficacy, multiple side effects and recurrence of attacks (7). Thereby, the need to identify alternative treatment modalities for IBD remains.

Sulforaphane (SFN), an isothiocyanate present in cruciferous vegetables such as broccoli and brussels sprouts, has attracted a lot of attention due to its unique ability to activate Kelch-like ECH-associated protein 1 (Keap1)–nuclear factor erythroid-2-related factor 2 (Nrf2)–antioxidant response elements (ARE) pathway (8). A variety of beneficial functions, such as anti-oxidant owned by SFN, rely on the induction of Nrf2-driven proteins. Most recently, there has been an increased focus on the anti-inflammatory and mucosa protective effects of SFN. Our previous results have confirmed that SFN significantly increased tight junction proteins expression both *in vitro* and *in vivo* models (9). In addition, SFN also exhibits a protective role against inflammation by notably decreasing the expression of various pro-inflammatory cytokines (9, 10). Furthermore, SFN has been shown to reverse the gut microbiota dysbiosis in mice, and also increased levels of intestinal short-chain fatty acids (9). Therefore, we hypothesized that SFN would be effective in ameliorating intestinal damage and dysfunction seen in IBD. The results from a previous study by Wagner et al. found that pretreatment with SFN at a dose of 25 mg/kg for 7 days significantly improved symptoms and reduced the inflammatory biomarker's expression in dextran sulfate sodium (DSS)-induced UC mice (11). Another study by Zhang et al. (12) reported that SFN reversed the gut dysbiosis and also reduced the damage in DSS-induced colitis mice. Thereby, in this study, we utilized a DSS-induced colitis model in mice to thoroughly investigate the protective effects and uncover the associated mechanism of SFN in treatment of IBD.

## Materials and Methods

### Materials and Chemicals

SFN was purchased from Toronto Research Chemicals (Toronto, Canada). DSS (36,000–50,000 Da molecular weight) was obtained from MP Biomedicals, Inc. (Solon, USA). Mesalazine was obtained from Ipsen Pharmaceutical Co. (France). NP40 lysis buffer, BCA protein assay kit, dimethylsulfoxide, and normal saline were purchased from the Beyotime Institute of Biotechnology (Nantong, China). Primary antibodies against ZO-1, Occludin, Claudin-1, Nrf2, UDP-glucuronosyltransferase (UGT), Signal Transducer, and Activator of Transcription-3 (STAT3), Cyclooxygenase-2 (COX-2), β-actin, and horseradish peroxidase (HRP)-conjugated secondary antibodies were obtained from Proteintech Group, Inc. (Wuhan, China). Mouse IL-6, interferon (IFN)-γ, TNF-α, and IL-1β enzyme-linked immunosorbent (ELISA) kits were obtained from Elabscience Biotechnology Co., Ltd (Wuhan, China). A Western blot enhanced chemiluminescence kit was purchased from Advansta, Inc. (San Jose, USA).

### Animal Experiment and Dosage Regimen

Seventy 8-week-old male C57BL/6 mice were purchased from Shanghai Slac Laboratory Animal Co. Ltd (Shanghai, China) and housed in the facilities of Laboratory Animal Services at Ningbo University (temperature 22 ± 2°C and humidity 55 ± 5% with a 12-h/12-h light/dark cycle). Mice were allowed to acclimate for 2 weeks prior to the experiment. The mice (nearly 25 g) were randomly assigned to eight groups (10 mice for the groups B–F; six mice for the group A; and seven mice for the groups G and H). DSS was dissolved in drinking water at a concentration of 2% DSS (w/v) to induce colitis. In the DSS treatment groups, mice received distilled water for 7 days, then 2% DSS for 7 days, followed by 5 days of distilled water. SFN was dissolved in a small volume of DMSO and diluted to the appropriate concentration with normal saline. The final concentration of DMSO did not exceed 1%. In the SFN-treated groups, mice were gavaged with SFN (2.5, 5, 10, and 20 mg/kg/day for the groups C–F, separately) during the whole experimental procedure. In the groups G and H, mice were orally administrated with Mesalazine (50 and 100 mg/kg/day) during DSS treatment time that served as a positive medical treatment group. All mice in each group had identical total gavage volume and identical contents of solvent.

**Figure 1:**
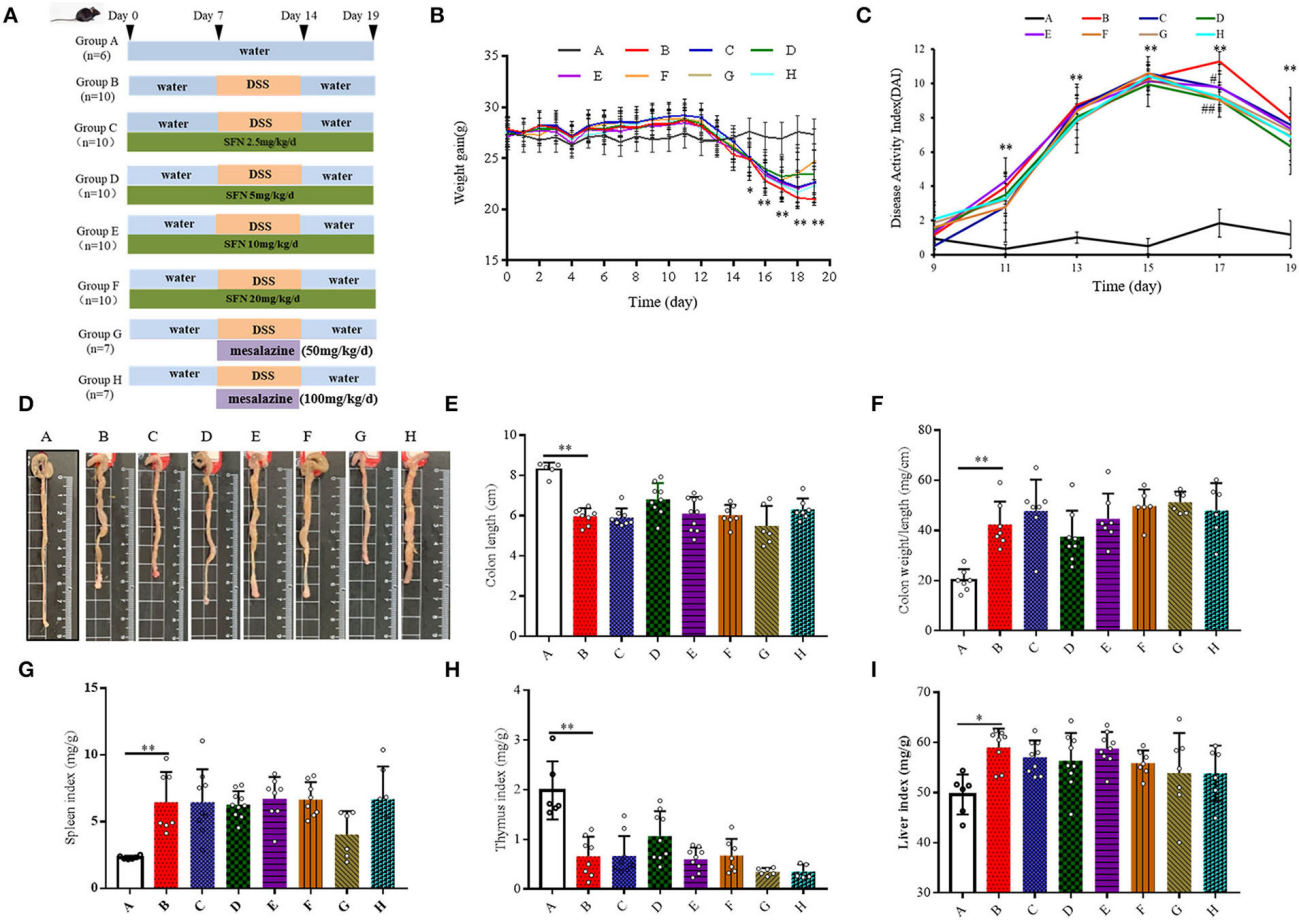
Effect of SFN on colitis symptoms in mice. **(A)** Scheme of the animal experimental design. Group A: Negative control; Group B: Colitis model (2% DSS in drinking water); Group C: SFN (2.5 mg/kg/d) + colitis model (2% DSS in drinking water); Group D: SFN (5 mg/kg/day) + colitis model (2% DSS in drinking water); Group E: SFN (10 mg/kg/day) + colitis model (2% DSS in drinking water); Group F: SFN (20 mg/kg/day) + colitis model (2% DSS in drinking water); Group G: Mesalazine (50 mg/kg/day) + colitis model (2% DSS in drinking water); Group H: Mesalazine (100 mg/kg/day) + colitis model (2% DSS in drinking water). **(B)** Mean body weight over time. **(C)** DAI score. **(D)** Representative macroscopic pictures of colons in each group. **(E)** Colon length. **(F)** Colon weight/ length ratio. **(G)** Spleen index. **(H)** Thymus index. **(I)** Liver index. All data are presented as the mean ± SD. *P* value < 0.05 was considered to indicate statistical significance (*P < 0.05 and **P < 0.01 compared with Group A, #*P* < 0.05 and ##*P* < 0.01 compared with Group B).

All mice were maintained in solid-bottom cages and were allowed free access to food and water during the entire experimental procedure. Body weight and consumption of food and water for all mice were monitored every day throughout the whole experimental procedure. Mouse survival was closely monitored throughout the experimental period. The whole experimental period lasted for 19 days. The experimental scheme is shown in Figure 1A. The study was approved by the Ethics Committee of Ningbo University (Registration Number: NBU20210028) and performed according to the Guidelines for Animal Care.

### Sample Collection and Disease Activity Index

DAIs of mice, including body weight loss (%), stool consistency, and blood in feces, were measured and recorded according to the DAI scoring system in Supplementary Table 1. DAI score, which is defined as the average of body weight loss score, fecal character score, and blood stool fraction, was used to evaluate the severity of colitis. At the end of the experiment, all of the mice were sacrificed and tissues (colon, spleen, thymus, and liver) were collected. Colon segments between the ileocecal junction and the anus were removed. Colons were weighed and washed with ice-cold PBS, and colonic contents were collected and stored at −80°C. Distal colon parts were fixed in 4% paraformaldehyde, embedded in paraffin, and stained with hematoxylin & eosin (H&E) to observe the tissue morphological changes with an optical microscope and conduct histological evaluation, while the other parts were stored at −80°C for further studies.

### Histological Assessment

Colon specimens were embedded in paraffin and cut into 4 μm sections for H&E staining. The slides were examined with an Olympus BX40 microscope (200× and 400× magnification). All the slides were grossly inspected in a blinded fashion by two pathological experts. The inflammatory scores are presented as the sum of the four parameters as shown in Supplementary Table 2.

### Determination of Cytokines Concentration in Serum

Blood samples from mice were collected by retro-orbital sinus puncture under isoflurane anesthesia *via* the medial canthus of the eye using clean heparinized microhematocrit tubes. Blood was collected and centrifuged at 3,000 rpm for 15 min, and then the serum was obtained. Aliquots of each serum sample were stored at −80°C. The concentrations of IL-6, IFN-γ, TNF-α, and IL-1β were determined using ELISA kits (Elabscience Biotechnology Co., Ltd, China) according to the manufacturer's instructions. The concentrations were spectrophotometrically quantified by measuring the absorbance at 450 nm. The data were measured in pg mL^−1^.

### DNA Extraction and 16S rRNA Gene Sequencing Analysis

Total genomic DNA was extracted from fecal samples using the E.Z.N.A.^®^ DNA Kit (Omega Bio-Tek, Norcross, GA, USA). DNA concentration was assessed using a Nanodrop (Thermo Scientific). DNA integrity and size were assessed using agarose gel electrophoresis, and all the samples that were showing adequate concentration and integrity were kept for further sequencing analysis. The V3–V4 variable regions of the 16S rRNA gene extracted from each fecal sample were amplified using primers 338F (5′-ACTCCTACGGGAGGCAGCA-3′) and 806R (5′-GGACTACHVGGGTWTCTAAT-3′) on a GeneAmp 9700 thermal cycler PCR system (Applied Biosystems, USA). PCR amplicons were purified using the AxyPrep DNA Gel Extraction Kit (Axygen, USA) and quantified using a QuantiFluor™-ST fluorometer (Promega, USA). After the individual quantification step, amplicons were pooled in equal amounts, and pair-end 2 × 300-base pair (bp) sequencing was performed using the Illumina MiSeq platform. Raw fastq files were demultiplexed and quality-filtered by the Quantitative Insights into Microbial Ecology (QIIME) platform and R packages (v3.2.0). Operational taxonomic units (OTUs) were picked using a criterion of 97% nucleotide identity. α-Diversity was measured by species richness and evenness from the rarefied OTU and indicated as the Shannon and Simpson indices. Sequencing data were analyzed using the free online Majorbio Cloud Platform (https://cloud.majorbio.com/).

### Gas Chromatography Analysis of Volatile Fatty Acids in the Samples of Colonic Contents

The whole procedure was performed as mentioned in the previous study (9). Briefly, the samples of colonic contents (1 g) from all the groups of mice were homogenized in 5 ml of deionized water for 10 min and then centrifuged at 13,200 × *g* for 20 min at 4°C. The supernatant was immediately filtered through a 0.45 μm microfiber filter. Then, 1 ml of supernatant was placed in a 1.5 ml GC vial, to which 100 μL of formic acid was added. Standard curves for seven fatty acids (99%, analytical standard, Sigma) were made to analyze the concentrations of volatile fatty acids from the colonic contents of the mice. Volatile fatty acids were quantified by GC (Agilent 7,890; Agilent Technologies, USA) equipped with a flame ionization detector (FID). The concentrations of total fatty acids were calculated as the sum of those of volatile fatty acids (acetic acid, propionic acid, butyric acid, iso-butyric acid, valeric acid, iso-valeric acid, and caproic acid).

### Protein Extraction and Western Blotting Analysis

Frozen colon samples were ground into powder in liquid nitrogen and lysed in ice-cold NP-40 lysis buffer containing 1 mM of protease inhibitor PMSF for 30 min. Colon tissue homogenate was centrifuged at 16,000×*g* for 20 min at 4°C, the supernatants were collected for Western blot analysis. Equal amounts of proteins (60 μg) were subsequently separated with SDS–polyacrylamide gel electrophoresis and transferred to PVDF membranes. After blocking with 5% skimmed milk at room temperature for 1 h, the membranes were incubated with primary antibodies at 4°C overnight, washed with TBST, and incubated with HRP-conjugated secondary antibodies at room temperature for 1.5 h. The membranes were visualized with an enhanced chemiluminescence reagent. The relative densities of the individual bands were analyzed densitometrically using the ChemiImager 4,000 instrument (Alpha Innotech, USA).

### Statistical Analyses

All data were reported as the mean ± standard deviation (SD). Statistical analysis was carried out using SPSS 19.0 software (SPSS Inc., Chicago, IL, USA) and Graphpad prism 6. For data sets confirmed with normal distribution (Shapiro-Wilk test), an unpaired Student's *t*-test was performed between Group A and Group B, and one-way ANOVA was performed to compare the effects of SFN and Mesalazine treatment in the DSS-treated group, followed by Dunnett's multiple comparison test against Group B. For the data sets that are not normally distributed, the Mann–Whitney test was performed between Group A and Group B, and the Kruskal-Wallis tests were performed for Groups B–H with Dunn's *post-hoc* test against Group B. The *P*-value generated from the multiple comparisons has already been adjusted by family-wise significance and confidence levels of 0.05.

## Results

### SFN-Alleviated DSS-Induced Colitis in Mice

Body weight was recorded at the same time point every day. As shown in Figure 1B and Supplementary Table 3, the body weight of all DSS treatment groups (i.e., the Groups B–F) decreased significantly on the 15^th^-19^th^ day. On the 19th day, 20 mg/kg/day of SFN (Group F) improved the body weight loss in contrast to Group B (*P* < 0.05). DAI scores were used to evaluate the severity of colitis (Figure 1C). On the 11th, 13th, 15th, and 19th day, the DAI scores were increased significantly in Group B than that in Group A. On the 17th day, the DAI scores in the SFN- and Mesalazine-treatment groups (groups D, F–H) were decreased significantly than that in Group B (*P* < 0.05). The detailed DAI scores at six different time points are shown in Supplementary Table 4.

As shown in Figure 1D, in Group B, characteristic macroscopic manifestations of DSS colitis-colon length shortening, hyperemia, and edema were observed. The colon length and weight/length ratio of mice in Group B shortened significantly in comparison to that in Group A (Figures 1E,F).

The spleen, thymus, and liver of mice from all groups were also measured at the end of the experiment (Figures 1G–I). Compared with Group A, the indices of the spleen and liver both increased, whereas the thymus index decreased significantly in Group B (*P* < 0.05). The survival rate and the consumption of food and water are shown in Supplementary Figures 1A–C.

### SFN Ameliorated Colonic Tissue Damage in Colitis Mice

**Figure 2:**
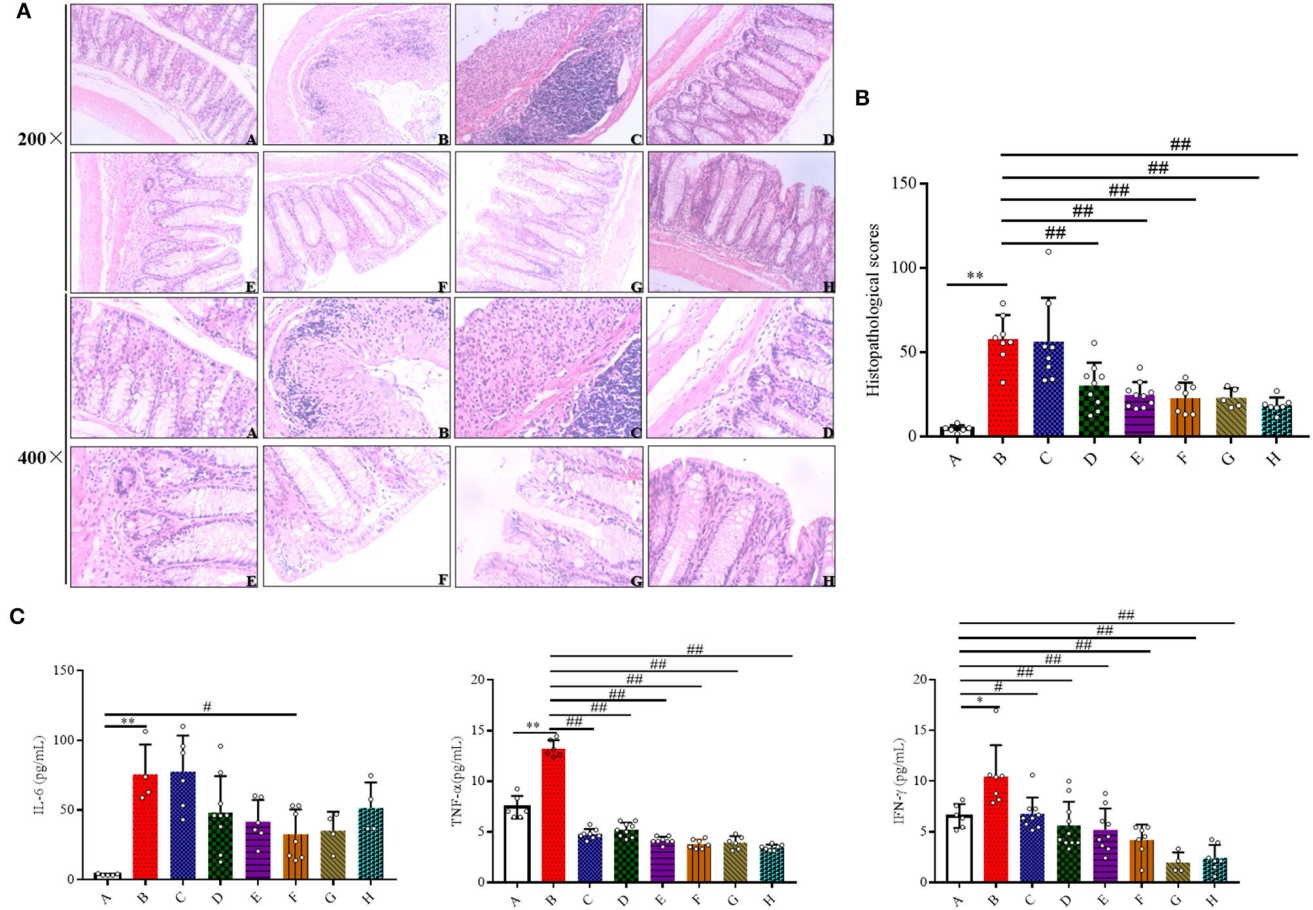
Effects of SFN on histopathological characteristics and inflammatory in colitis mice. **(A)** Representative H&E staining of colon tissues (magnification ×200, and ×400). **(B)** Colonic histological score. **(C)** Effects of SFN on the concentrations of IL-6, TNF-α, and IFN-γ in plasma were determined by ELISA at a concentration of pg/ml. All data are presented as the mean ± SD. *P-*value < 0.05 was considered to indicate statistical significance (*P < 0.05 and **P < 0.01 compared with Group A, ^#^*P* < 0.05 and ^*##*^*P* < 0.01 compared with Group B).

The protective effects of SFN on histological damages in the colons were examined. Compared with Group A, the colonic specimens obtained from Group B were characterized by significant loss of normal crypts, alteration of epithelial structure, increase of neutrophil and lymphocyte infiltration into the mucosal and submucosal layers, extensive loss of glands, and severe lesions in the colon mucosa, which collectively resulted in a significant higher histological score (Figures 2A,B). As observed in Group B, 5, 10, and 20 mg/kg/day of SFN and Mesalazine treatment (50 and 100 mg/kg) remarkably mitigated the morphological alterations and protected the colonic tissue integrity, which ultimately lead to a significant decrease in the total histological score. However, 2.5 mg/kg/day of SFN treatment showed more severe morphological damages in colonic specimens, resulting in a higher histological score.

### SFN Inhibited Inflammatory Cytokines Production in Colitis Mice

To evaluate if the SFN treatment could ameliorate colitis by modulating the inflammatory response, inflammatory cytokines were measured. As shown in Figure 2C, the concentrations of IL-6 were increased in the DSS-treated groups when compared with the negative control group. A 20 mg/kg/day of SFN treatment decreased IL-6 concentration in Group B (30.04 ± 18.07 vs. 75.49 ± 21.60 pg/ml, *P* < 0.05). TNF-α production was increased significantly in Group B in contrast with Group A (7.44 ± 1.12 vs. 13.24 ± 0.83 pg/mL, *P* < 0.05). TNF-α concentrations were decreased significantly in the SFN- and Mesalazine-treatment groups when contrasted with Group B (*P* < 0.01). IFN-γ concentration was increased significantly in Group B than that in the control group (6.57 ± 1.18 vs. 10.48 ± 3.78 pg/ml, *P* < 0.05). In the SFN- (2.5, 5, 10, and 20 mg/kg/day) and Mesalazine-treatment groups, the concentrations of IFN-γ were decreased significantly when compared with Group B (*P* < 0.05). IL-1β concentrations were slightly increased in Group B although no significant difference has been observed (data not shown).

### SFN-Modulated Gut Microbiota Composition

Given the critical role of gut microbiota in the pathogenesis of colitis, the effect of SFN on the state of gut microbiota composition in DSS-treated mice was investigated. The influences of SFN on the richness and evenness of gut microbiota were assessed. OTU numbers, Shannon diversity, Simpson, ACE, and Chao 1 indices were measured as they are common measures of α diversity that indicate the depth of sequence coverage and community diversity. As shown in Supplementary Figures 1D,E, there were no significant differences among all the groups at α-diversity.

**Figure 3:**
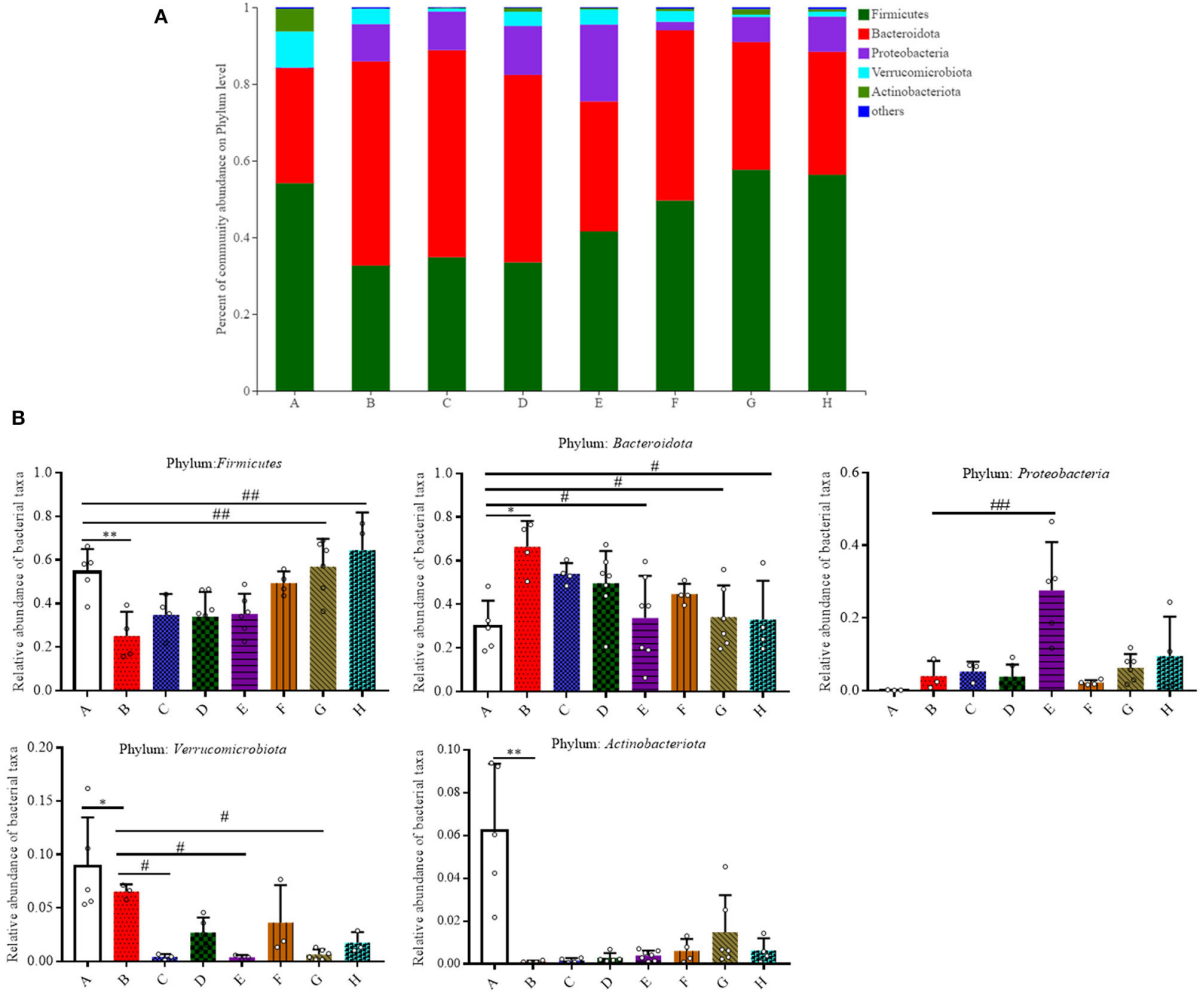
Effect of SFN on gut microbiota compositions at the phylum level in colitis mice. **(A)** Gut microbiota compositions at the phylum level in each group. **(B)** Relative abundance of predominant bacterial community members at the phylum level. All data are presented as the mean ± SD. *P*-value <0.05 was considered to indicate statistical significance (*P < 0.05 and **P < 0.01 compared with Group A, ^#^*P* < 0.05 and ^*##*^*P* < 0.01 compared with Group B).

The relative abundance of the predominant taxa among groups was evaluated (Figures 3A,B). *Firmicutes, Bacteroidota, Proteobacteria, Verrucomicrobiota*, and *Actinobacteriota* were five major phyla that account for more than 95.0% of the whole gut microbial composition in all the mice. As shown in Figure 3B, at the phylum level, the relative abundance of *Firmicutes* was significantly decreased in Group B when compared to Group A (0.54 ± 0.10 vs. 0.25 ± 0.11, *P* < 0.05). In comparison to Group A, *Bacteroidota* were elevated in Group B (0.30 ± 0.12 vs. 0.66 ± 0.12, *P* < 0.05). SFN (10 mg/kg/day) and Mesalazine treatment was shown to significantly lower the relative abundance of *Bacteroidota* in contrast with the counterparts in Group B (*P* < 0.05). DSS-treated mice with 10 mg/kg/day SFN had a relative higher abundance of *Proteobacteria* when compared to Group B (*P* < 0.01). In comparison to Group A, the relative abundance of *Verrucomicrobiota* was decreased in Group B (*P* < 0.05). While the results in the SFN—(2.5 and 10 mg/kg/day) and Mesalazine-treatment (50 mg/kg/day) groups had a relative lower abundance of *Verrucomicrobiota* in contrast with Group B (*P* < 0.05). The abundance of *Actinobacteriota* in Group B was significantly decreased than that in Group A (*P* < 0.05).

**Figure 4:**
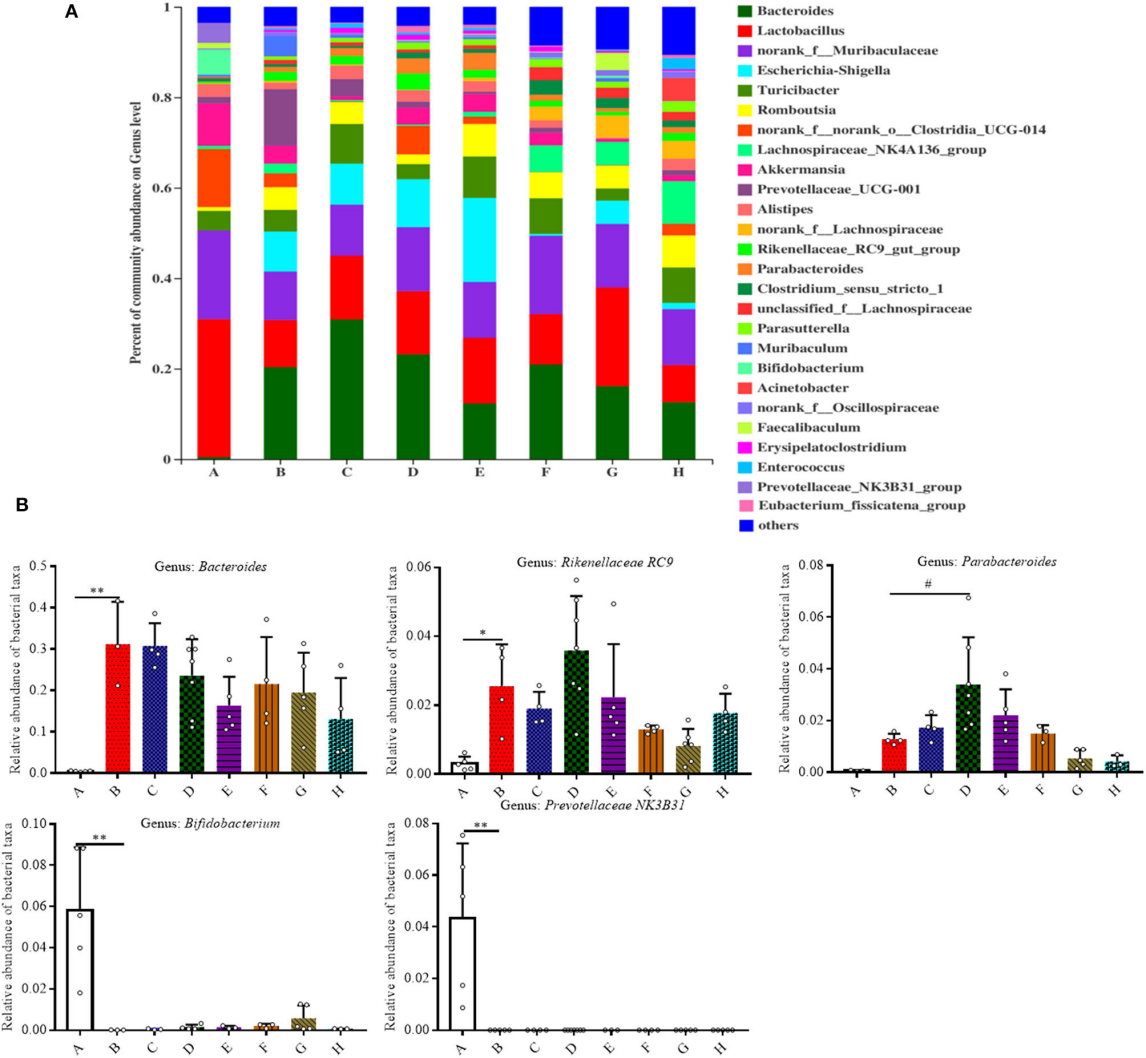
Effect of SFN on gut microbiota compositions at the genus level in colitis mice. **(A)** Gut microbiota compositions at the genus level in each group. **(B)** Relative abundance of predominant bacterial community members at the genus level. All data are presented as the mean ± SD. *P*-value <0.05 was considered to indicate statistical significance (*P < 0.05 and **P < 0.01 compared with Group A, ^#^*P* < 0.05 and ^*##*^*P* < 0.01 compared with Group B).

We then analyzed the relative abundance of bacteria at the genus level (Figures 4A,B). Among all the treatment groups, *Bacteroides, Rikenellaceae RC9, Parabacteroides, Bifidobacterium*, and *Prevotellaceae NK3B31* were the most affected genera. In comparison to Group A, the relative abundance of *Bacteroides* and *Rikenellaceae RC9* was significantly increased in the DSS group (*P* < 0.05). The relative abundance of *Parabacteroides* was significantly increased in Group D in contrast with Group B (*P* < 0.05). In contrast with Group A, the relative abundance of *Bifidobacterium* and *Prevotellaceae NK3B31* was significantly decreased in Group B (*P* < 0.01).

**Figure 5:**
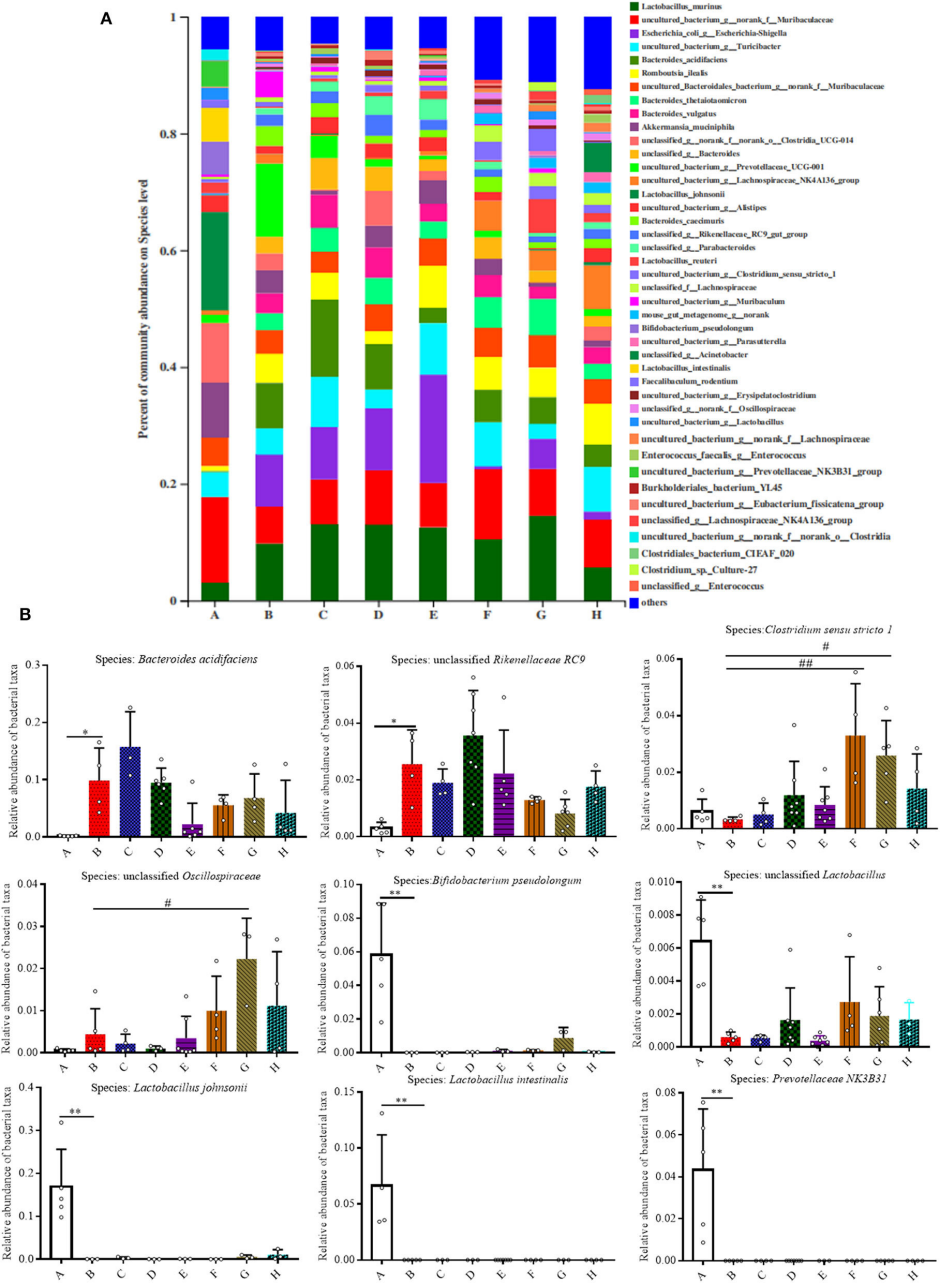
Effect of SFN on gut microbiota compositions at the species level in colitis mice. **(A)** Gut microbiota compositions at the species level in each group. **(B)** Relative abundance of predominant bacterial community members at the species level. All data are presented as the mean ± SD. *P*-value <0.05 was considered to indicate statistical significance (*P < 0.05 and **P < 0.01 compared with Group A, ^#^*P* < 0.05 and ^*##*^*P* < 0.01 compared with Group B).

As shown in Figures 5A,B, the relative abundances of bacteria at the species level were also analyzed for all the groups, and it was found that 42 species accounted for more than 90% of the fecal pellet. Of these detected species there were nine species that showed significant changes. In comparison to Group A, the relative abundances of *Bacteroides acidifaciens* and *Rikenellaceae* RC9 both increased in Group B (*P* < 0.05). Meanwhile, the relative abundances of *Bifidobacterium pseudolongum, unclassified Lactobacillus, Lactobacillus johnsonii, Lactobacillus intestinalis*, and *uncultured Prevotellaceae NK3B31* in Group B were all significantly lower than that in Group A (*P* < 0.01). The relative abundance of *Akkermansia mucinphila* was lower in Group B than that in Group A, although no significant difference is shown (Supplementary Figure 2).

### SFN Promoted the Production of Volatile Fatty Acids

**Figure 6:**
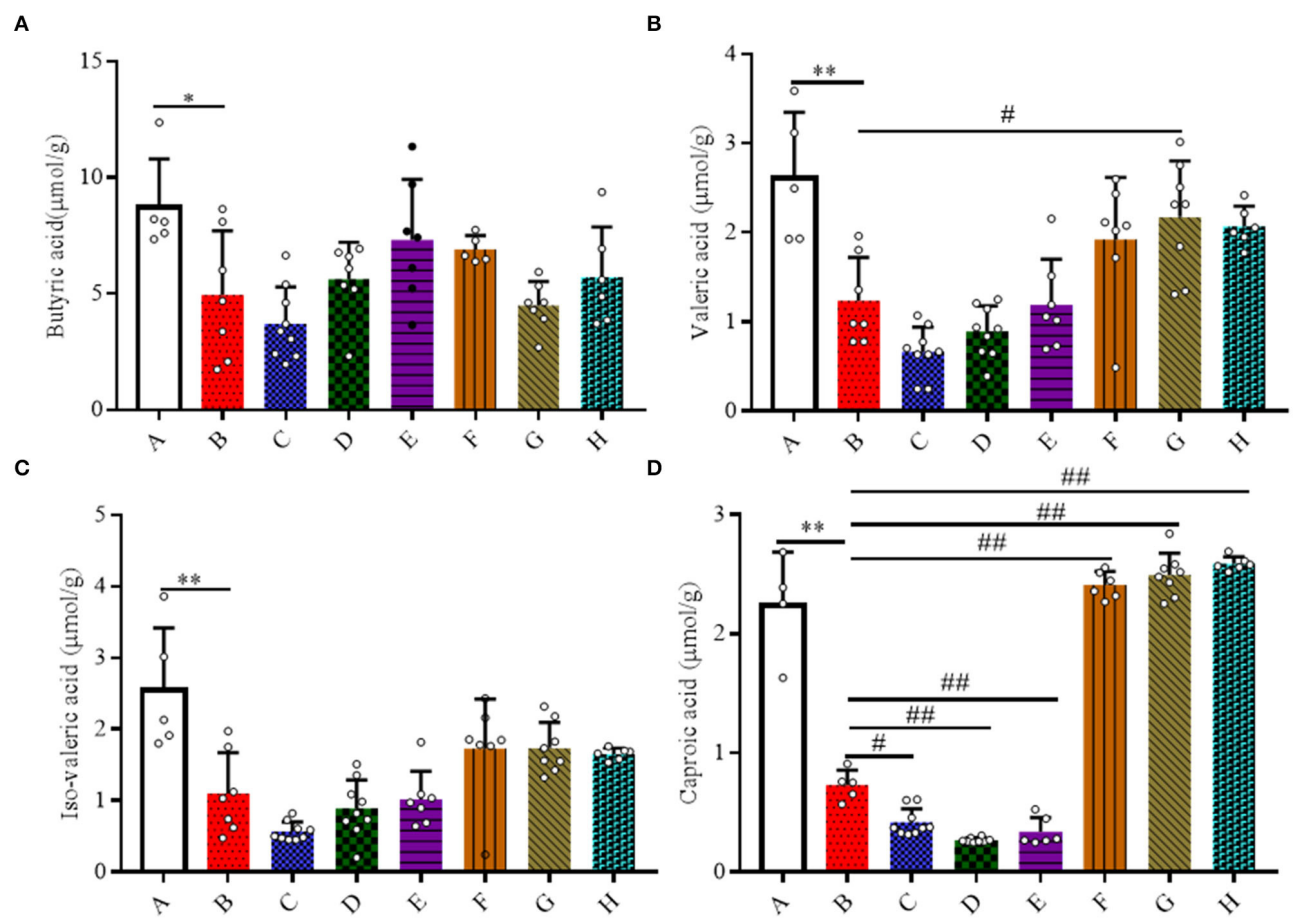
Effect of SFN on volatile fatty acids concentrations in colitis mice. The samples were collected from colonic contents of all groups of mice. **(A)** Butyric acid; **(B)** Valeric acid; **(C)** Iso-valeric acid; **(D)** Caproic acid. All data are presented as the mean ± SD. *P-*value <0.05 was considered to indicate statistical significance (*P < 0.05 and **P < 0.01 compared with Group A, ^#^*P* < 0.05 and ^*##*^*P* < 0.01 compared with Group B).

Here, the quantities of SCFAs (acetic acid, propionic acid, butyric acid, iso-butyric acid, valeric acid, and iso-valeric acid) and caproic acid in the colonic contents of mice were measured (Figure 6). A 20 mg/kg/day dose of SFN significantly increased the concentration of caproic acid in contrast with Group B (*P* < 0.01). For the levels of acetic acid, propionic acid, iso-butyric acid, and total fatty acids, there were no obvious differences among all the groups (Supplementary Figure 3).

### Correlation Analysis

**Figure 7:**
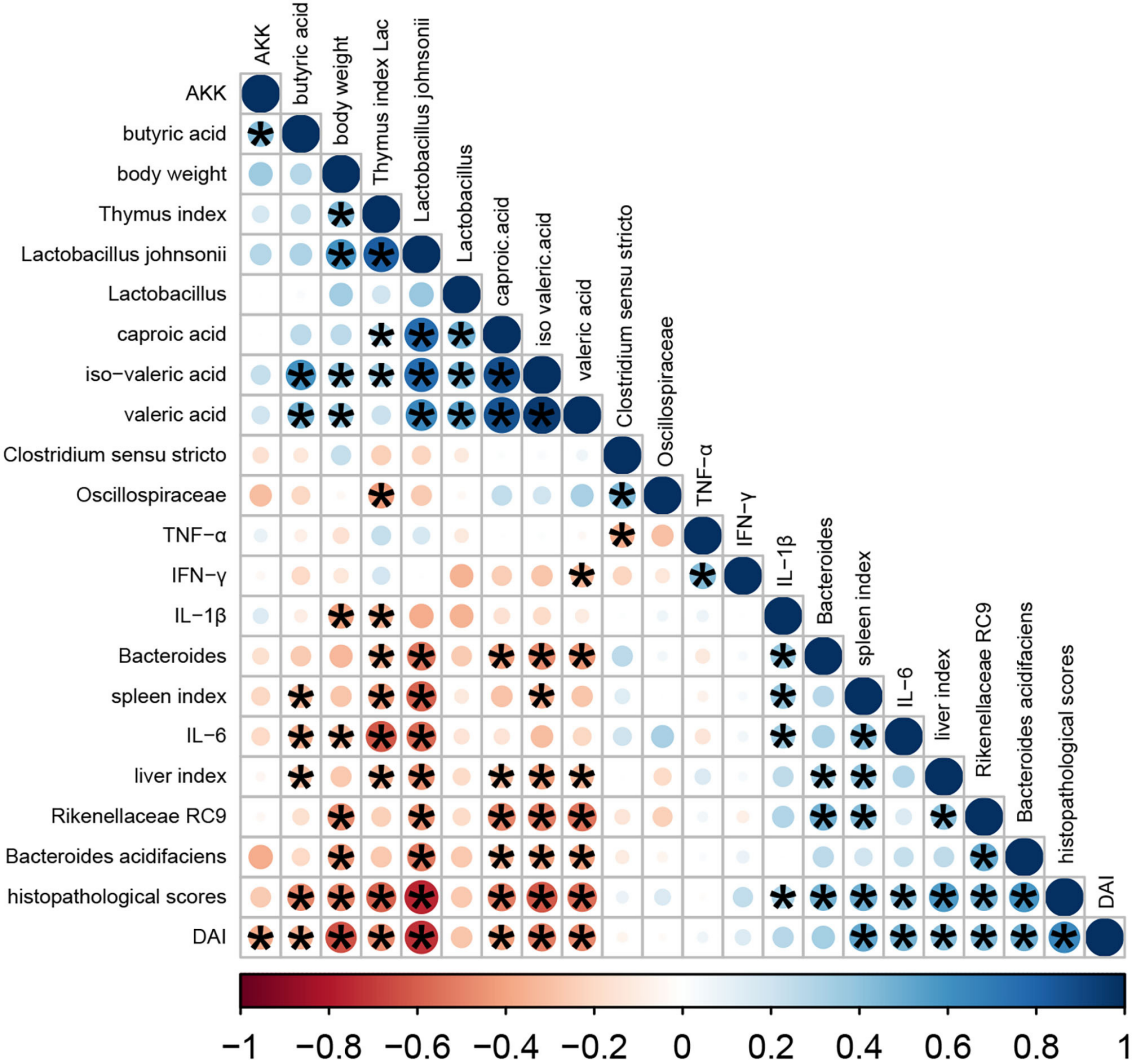
Heatmap of Pearson correlation analysis. The color scale bar ranged from −1.0 (red) to 1.0 (blue). The size and color of the circles indicate the magnitude of the correlation between parameters. Red and blue, respectively, denote negative and positive correlations. These were done in R.

In this study, we performed a correlation analysis to evaluate the statistical relationship among microbial species at the genus level, volatile fatty acids, and phenotype indicators (namely, organ indexes, DAI, body weight, and histopathological score) (Figure 7). Heatmap analyses revealed that the histopathological score had a negative correlation with body weight, thymus index, and volatile fatty acids (mainly, butyric acid, valeric acid, iso-valeric acid, and caproic acid) and *Lactobacillus johnsonii* (*P* < 0.05). Meanwhile, histopathological score had a positive correlation with DAI, spleen index, liver index, proinflammatory cytokines, *Bacteroides acidifaciens*, unclassified *Rikenellaceae RC9*, and unclassified g *Bacteroides* (*P* < 0.05).

### SFN Upgraded Tight Junction Protein Expression

**Figure 8:**
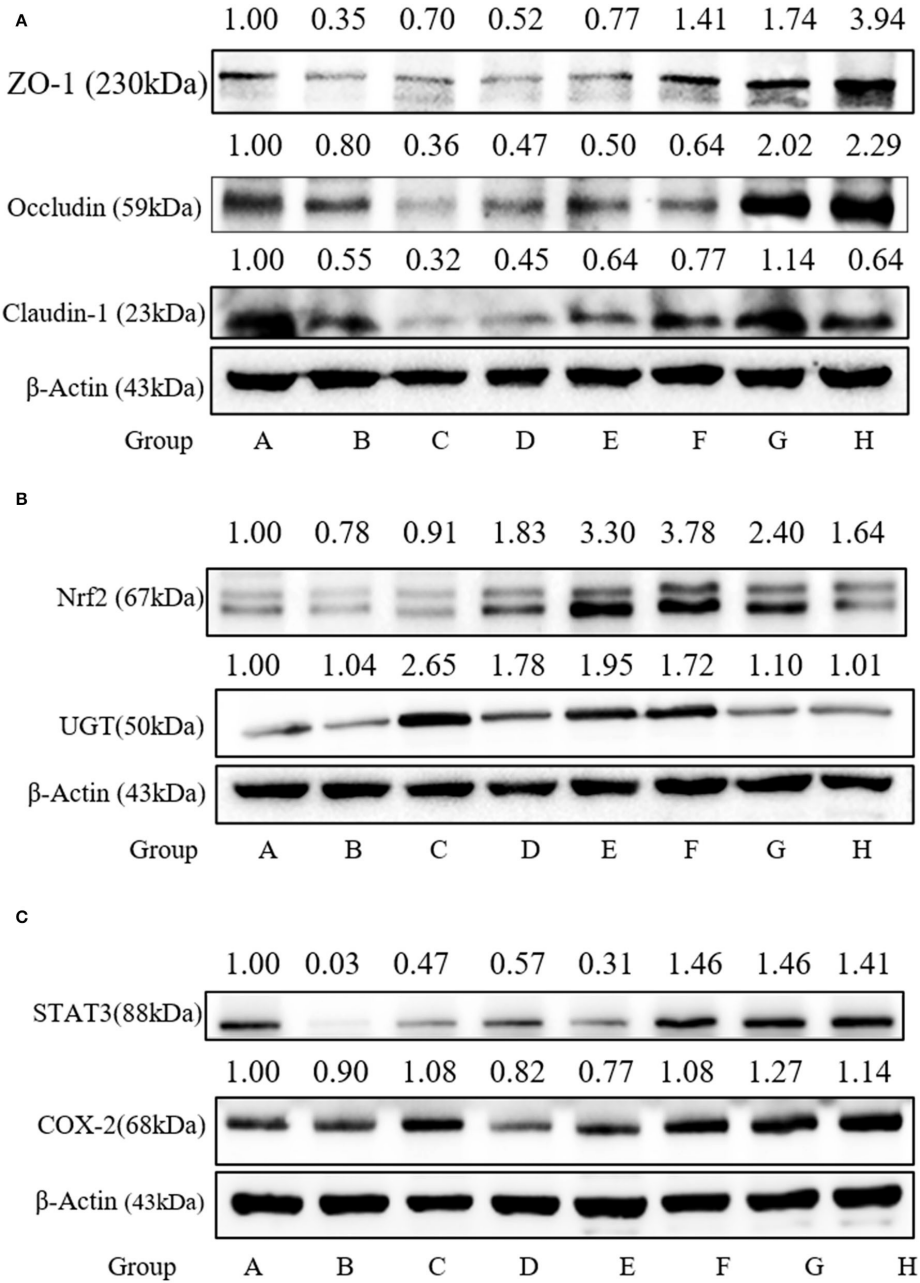
Effect of SFN on tight junction and Nrf2-related protein expression in colitis mice. **(A)** Relative expression of ZO-1, Occludin, and Claudin-1 in colon tissues as measured by Western blotting. **(B)** Relative expression of Nrf2 and UGT in colon tissues as measured by Western blotting. **(C)** Relative expression of STAT3 and COX-2 in colon tissues as measured by Western blotting. All data are presented as the mean ± SD.

To confirm the protective effect of SFN against the gut barrier disruption in colitis mice, tight junction proteins, including ZO-1, Claudin-1, and Occludin, serving as the basis of structure for the paracellular permeability barrier, were measured. As shown in Figure 8A, the expressions of tight junction proteins were all decreased in Group B. The expression of ZO-1 in Group F was increased significantly when compared to Group A (*P* < 0.05).

### SFN-Regulated Nrf2-Related Protein Expression

SFN exerts multi-functional effects *via* the activation of Nrf2 antioxidants responsive element pathways. Nrf2 can regulate the expression of more than 200 genes, such as phase 2 enzymes and STAT3. Here, the expressions of Nrf2 and UGT in the colonic species were measured. As shown in Figure 8B, the Nrf2 expression was increased significantly by 5, 10, and 20 mg/kg/day of SFN treatment. As shown in Figure 8C, STAT3 expression is decreased by 0.03-fold in the colitis group. SFN- and Mesalazine treatment increased the STAT3 expression significantly when compared to Group B. COX-2, an important enzyme in the synthesis of prostaglandin from arachidonic acid, is inducible in response to cytokines. In the present study, there were no significant changes among all the groups for COX-2 expression (Figure 8C).

## Discussion

In this study, the utilization of 2% DSS successfully established a mice colitis model, in mice according to Stefan's protocol, as evidenced by decreased body weight, reduced food intake, shortened colon length, and increased DAI scores (13). In comparison to Group B, mice treated with SFN presented with weight gain, decreased DAI score, and colon length growth. Moreover, histological results clearly showed that SFN ameliorated pathological damage in the colitis mice. These present findings strongly indicate that treatment with SFN is highly effective against DSS-induced colitis. The potential mechanisms were also explored, including the suppression of inflammation, improvement of intestinal mucosa barrier defects, and modulation of microbiota dysbiosis.

Cytokines have been directly implicated in the pathogenesis of IBD in recent studies, and they have been established as playing a pivotal role in aggravating and controlling intestinal inflammation and the associated clinical symptoms of IBD (14). The results from H&E staining analysis clearly showed obvious infiltration of inflammatory cells, multiple erosions, and ulcers in the colon tissue of DSS treated mice. Three critical pro-inflammatory cytokines - IL-6, TNF-α and IFN-γ are known to be predominantly involved in IBD. IL-6 production by lamina propria macrophages and CD4^+^ cells is increased in the experimental colitis and in IBD patients (15). Additionally, Robust IFN-γ production has been observed in colitis in mice and IBD patients (16). A study using *IFN-*γ^−/−^ mice showed significantly decreased inflammation in DSS colitis, indicating an indispensable role of IFN-γ in colitis initiation (17). Furthermore, anti-TNF antibodies are now successfully used in the therapy of IBD, suggesting its pivotal role in the initiation and progression of IBD (18). In this present study, 5, 10, and 20 mg/kg/day of SFN treatment significantly ameliorated the inflammatory damage in mice colon tissue when compared to the colitis group, which is similar to the effect in the Mesalazine-treatment group. Accordingly, the increased concentrations of pro-inflammatory cytokines in the colitis mice, such as IL-6, IFN-γ, and TNF-α, were all significantly decreased by SFN treatment. Therefore, SFN treatment can alleviate IBD by decreasing the secretion of inflammatory cytokines.

We further analyzed inflammation-related molecules that are implicated in the etiology of UC. STAT3, a pleiotropic transcription factor, is an essential mediator of epithelial repair and inflammatory processes in colitis and colitis-associated colon cancer. In animal models, loss of STAT3 leads to more severe chronic inflammation following acute injury (19, 20). STAT3 activation in intestinal epithelial cells is essential for mucosal wound healing through its ability to regenerate epithelium, thereby playing an integral role in recovery from colitis (21). In this present study, the expression of STAT3 in the DSS-induced colitis mice was markedly decreased and significantly elevated when treated with SFN.

Gut microbiota dysbiosis closely relates to the pathogenesis of IBD. Our previous results have shown that SFN has the ability to modulate the gut microbiota (9). Typically, gut microbiota in IBD patients is characterized with a decrease in the commensal and beneficial fecal bacteria such as *Firmicutes*, but an increase in the inflammatory strains such as *Proteobacteria*, which is consistent with our findings in colitis mice (22, 23). As shown in Figures 3A,B, the DSS administration significantly decreased the relative abundance of *Firmicutes*, and this trend was reversed by treatment with SFN and Mesalazine. *Clostridium sensu stricto, Oscillospiraceae*, and *Prevotellaceae*, belonging to *Firmicutes* phylum, are known to produce butyrate from their fermentable carbon sources (24, 25). As shown in Figure 5, SFN treatment significantly increased the abundance of *Clostridium sensu stricto* 1 and *unclassified Oscillospiraceae*.

It has been reported that the patients with IBD have shown an increased number of *Bacteroidota* compared to that of the controls (26). In this present study, the relative abundance of *Bacteroidota* was increased significantly in the colitis mice, whereas it was decreased significantly by SFN and Mesalazine treatment. According to the results at the genus and species level, the increased abundance of *Bacteroidota* should be attributed to *Bacteroides acidifaciens* and unclassified *Bacteroides*. *Bacteroides acidifaciens* leads to the progression and development of colitis (27). *Bacteroides acidifaciens* has several features, for example, degrading mucin, which is a protective layer in the colon produced by epithelial layers. Moreover, *Bacteroides acidifaciens* is also known to increase acetic and succinic acid production, both of which can contribute to colitis-associated inflammation (27). Notably, SFN treatment decreased this abundance of *Bacteroides acidifaciens* in DSS mice. *Rikenellaceae RC9* belongs to the *Rikenellaceae* family, *Bacteroidota* phylum. It is reported that *Rikenellaceae RC9* is significantly positively correlated with systemic inflammatory cytokines, such as IL-6,IL-1β,and TNF-α (28).

We noticed decreased relative abundances of both *Verrucomicrobiota* and *Actinobacteriota* in the colitis mice. The abundance of *Akkermansia muciniphila*, the only cultivated intestinal representative of the *Verrucomicrobiota*, was decreased in the DSS-induced mice. However, this decreased trend for *Akkermansia muciniphila* was reversed by SFN treatment. *Akkermansia muciniphila*, a Gram-negative and strictly anaerobic bacterium, is an important mucus-degrading intestinal bacterium that encodes mucin-degrading enzymes by increasing the thickness of the gut mucus. The abundance of *Akkermansia muciniphila* is known to be markedly reduced in IBD patients as compared with the abundance in healthy individuals (29). *Akkermansia muciniphila* administration improved DSS-induced colitis *via* its protective effect on the gut barrier and its ability to reduce inflammatory cytokines (30). *Actinobacteriota* are Gram positive, non-motile, non-spore-forming, anaerobic bacteria with multiple branching rods. *Bifidobacterium*, one of the most present *Actinobacteriota* in the human gut, have beneficial effects on the maintenance of the gut barrier because of its ability to produce SCFAs, especially butyric acid (31). As shown in Figure 5, the genus of *Bifidobacterium pseudolongum*, belonging to *Bifidobacterium* species, was significantly decreased in DSS-induced colitis mice.

*Lactobacilli* are important members of the commensal flora within intestinal tract. They are generally considered non-pathogenic and recognized as probiotics with ability to modulate host immune responses with function of strengthening the epithelial junction complexes, decreasing the pro-inflammatory cytokines, and increasing the anti-inflammatory cytokine production (32). A clinical trial of *Lactobacillus* found it to be moderately effective in ameliorating colitis symptoms (33). Here, the relative abundances of unclassified *Lactobacillus, Lactobacillus johnsonii*, and *Lactobacillus intestinalis* were all significantly decreased in colitis mice than controls. Heatmap analysis demonstrated the close connection of this disrupted microbial flora with disease severity, especially *Lactobacillus johnsonii, Bacteroides acidifaciens, Rikenellaceae RC9*, and unclassified *Bacteroides*. Further studies are needed to confirm these relationships and mechanistic role of these bacteria in IBD.

Bacteria in the gut are known to produce short-chain fatty acids along with medium- and long-chain fatty acids as end products of metabolism. The contents of butyric acid, iso-butyric acid, valeric acid, and iso-valeric acid were all decreased in DSS-induced colitis mice and in 2.5 mg/kg/day of the SFN treatment group, whereas this decreased tendency was reversed by 10 and 20 mg/kg/day of SFN. Recent studies regarding SCFAs, have highlighted their protective effects on various systems *in vivo* and *in vitro*. SCFAs, especially butyric acid, are quickly absorbed and utilized as a major energy source by intestinal epithelial cells with multiple beneficial effects on the host, from improving barrier function to attenuating inflammatory response to immune-regulatory effect (34). Butyrate administration can markedly ameliorate the inflammatory response and maintain the epithelium barrier integrity in mice with colitis. Our previous results have shown that SFN possesses the ability of increasing the quantities of butyric acid and iso-butyric acid in mice (9). Valeric acid ameliorates pro-inflammatory cytokine production and improves gastrointestinal tract function and intestinal epithelial integrity (9, 35). It is reported that the contents of propionic acid, butyric acid, iso-butyric acid, and valeric acid evidently decreased in the colonic contents in DSS-induced colitis mice, which is quite consistent with our results (36). Caproic acid shows anti-inflammatory effect for its reduction in NF-κB transactivation and decrease in inflammatory cytokine production (37, 38). More importantly, fecal levels of caproic acid have been shown to inversely correlate with Crohn's disease activity (39). Here, our results show that the colonic contents of caproic acid were significantly decreased in DSS-induced colitis mice and in 2.5 mg/kg/day of the SFN treatment group. The concentrations of caproic acid were markedly increased by 20 mg/kg/day of SFN treatment.

Disrupted intestinal epithelial barrier is an important characteristic of IBD. The paracelluar permeability of the epithelial barrier and their function in the colon are primarily governed by tight junction proteins, such as ZO-1, Occludin, and Claudin-1 (40). As shown in Figure 8A, DSS administration decreased expression of ZO-1, and SFN and Mesalazine treatment reversed this tendency. Our previous results from both *in vitro* and *in vivo* models also suggest that SFN upregulated the tight junction protein expression (9).

Numerous studies suggested that a variety of beneficial functions owned by SFN relied on the induction of Nrf2 and Nrf2-driven proteins. Our results here clearly show that SFN significantly increase the expression of Nrf2 and the representative Phase II enzyme UGT. It has been shown that Nrf2-deficient mice were more susceptible to DSS-induced colitis (41). Many agents involved with Nrf2 activation, have been found to improve DSS-induced colitis, establishing evidence for a Nrf2-dependent mechanism as a promising strategy for treating UC (42, 43).

## Conclusion

**Figure 9:**
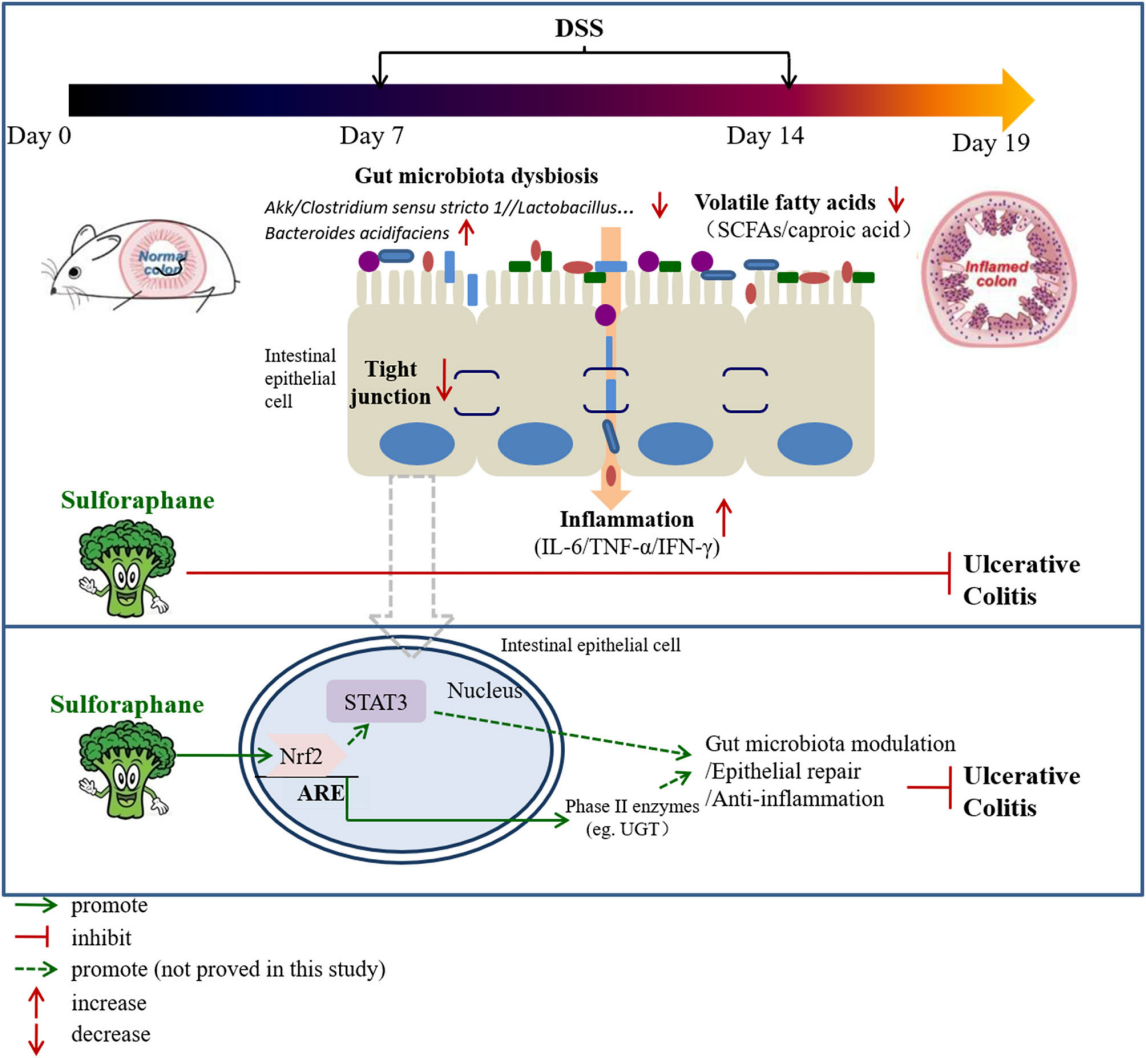
A proposed mechanism by which SFN protects against colitis.

Our data establishes evidence that SFN ameliorates UC through modulation of gut microbiota composition, increasing the contents of fecal volatile fatty acids (especially caproic acid), increasing expression of tight junction proteins, and reduction of pro-inflammatory cytokines (Figure 9). Nrf2 activation followed by STAT3 signaling pathway play a pivotal role in the protective effect of SFN on colitis. Therefore, SFN can be considered a potential candidate in the treatment of IBD.

## Data Availability Statement

The datasets presented in this study can be found in online repositories. The names of the repository/repositories and accession number(s) can be found below: NCBI [Accession: PRJNA815035].

## Ethics Statement

The animal study was reviewed and approved by Ethics Committee of Ningbo University (registration number: NBU20210028) and performed according to the Guidelines for Animal Care.

## Author Contributions

CH performed experiments, wrote and edited the manuscript, and contributed to funding acquisition. MG and HY performed experiments. XZ and PL analyzed the data. YQ and LZ partly funded the acquisition. All authors contributed to the article and approved the submitted version.

## Funding

This work was financially supported by the projection form the National Natural Science Foundation of China (No. 82103819), Natural Science Foundation of Zhejiang Province (Nos. LQ20H260007 and LY21H260001), and Natural Science Foundation of Ningbo City (Nos. 2021J125 and 202003N4202).

## Conflict of Interest

The authors declare that the research was conducted in the absence of any commercial or financial relationships that could be construed as a potential conflict of interest.

## Publisher's Note

All claims expressed in this article are solely those of the authors and do not necessarily represent those of their affiliated organizations, or those of the publisher, the editors and the reviewers. Any product that may be evaluated in this article, or claim that may be made by its manufacturer, is not guaranteed or endorsed by the publisher.
