# Supplementary material for: The Protective Effect of Sulforaphane on Dextran Sulfate Sodium-Induced Colitis Depends on Gut Microbial and Nrf2-Related Mechanism
Source: Front Nutr. 2022 Jun 27;9:893344. doi: 10.3389/fnut.2022.893344 (PMC9271993; doi:10.3389/fnut.2022.893344)
Supplement: Supplementary file 1 [file Data_Sheet_1.doc]

**The Protective Effect of Sulforaphane on dextran sulfate sodium-induced colitis depends on gut microbial and Nrf2 related mechanism**

**Supplement Table 1.** The disease activity index scoring criteria

| Score | Weight loss (%) | Stool consistency ^a^ | Bloody stool |
| --- | --- | --- | --- |
| 0 | None | Normal stool | Normal Stool |
| 1 | 1-5 | - | - |
| 2 | 6-10 | Loose stool | Feces occult blood |
| 3 | 11-20 | - | - |
| 4 | >20 | Watery diarrhea | Gross bleeding |

a: Normal stool: well-formed pellets. Loose stool: semi-formed stools which do not stick to the anus. Watery diarrhea: liquid stools sticking to the anus.

**Supplement Table 2.** The histologic inflammatory scores

| Score | Degree of Inflammatory cell infiltration (a) | Deepness of inflammatory cell infiltration (b) | Epithelial damage (c) | Extent of lesions (d) |
| --- | --- | --- | --- | --- |
| 0 | None | None | None | None |
| 1 | Mild | Mucosa | Disruption of architectural structure | Punctuate |
| 2 | Moderate | Mucosa and submucosa | Erosion | Multifocal |
| 3 | Severe | Transmural | Ulceration | Diffuse |

Total score=score (a)+score (b)+score (c)+ score (d).

**Supplement Table 3.** The body weight of mice in each group

| Group | D15 | D16 | D17 | D18 | D19 |
| --- | --- | --- | --- | --- | --- |
| A | 27.6±1.35 | 27.07±1.47 | 26.93±1.58 | 27.57±1.58 | 27.33±1.57 |
| B | 24.91±1.35^*^ | 22.76±1.19^**^ | 21.97±0.94^**^ | 21.09±1.07^**^ | 20.99±0.60^**^ |
| C | 25.07±0.98 | 23.60±0.92 | 22.76±1.04 | 22.16±1.50 | 22.63±1.60 |
| D | 25.07±1.89 | 23.91±1.91 | 23.19±2.01 | 23.39±2.16 | 23.47±2.33 |
| E | 24.83±1.61 | 23.34±1.27 | 22.56±1.59 | 22.02±1.75 | 22.57±1.98 |
| F | 24.86±2.02 | 23.32±1.97 | 22.85±2.04 | 23.51±1.33 | 24.66±1.71^#^ |
| G | 24.87±1.22 | 23.33±1.56 | 22.41±2.16 | 21.93±2.81 | 22.00±3.24 |
| H | 24.94±1.60 | 23.27±1.52 | 22.41±1.26 | 21.69±1.50 | 22.27±1.58 |

**Supplement Table 4.** The disease activity index scores of mice in each group

| Group | Day9 | Day11 | Day13 | Day15 | Day17 | Day19 |
| --- | --- | --- | --- | --- | --- | --- |
| A | 0.92±0.58 | 0.33±0.26 | 1.00±0.32 | 0.50±0.45 | 1.83±0.82 | 1.17±0.82 |
| B | 1.15±0.85 | 3.95±0.80^**^ | 8.75±0.54^**^ | 10.30±0.54^**^ | 11.30±0.57^**^ | 7.89±1.88^**^ |
| C | 0.50±0.47 | 2.80±1.36 | 8.60±0.70 | 10.60±0.52 | 9.75±1.36 | 7.58±1.47 |
| D | 1.50±0.82 | 3.50±1.08 | 8.05±0.76 | 9.95±1.30 | 9.05±1.01^##^ | 6.30±1.60 |
| E | 1.30±0.82 | 4.30±1.36 | 8.50±0.85 | 10.15±0.58 | 9.80±0.76 | 7.35±0.71 |
| F | 1.61±0.86 | 2.78±2.05 | 8.39±1.56 | 10.61±0.96 | 9.00±0.50^##^ | 6.93±0.93 |
| G | 1.86±1.03 | 3.21±0.95 | 7.79±1.35 | 10.21±0.86 | 9.25±0.66^##^ | 7.21±1.95 |
| H | 2.07±1.02 | 3.29±1.44 | 7.86±1.91 | 10.43±0.61 | 9.20±0.45^#^ | 6.86±1.38 |


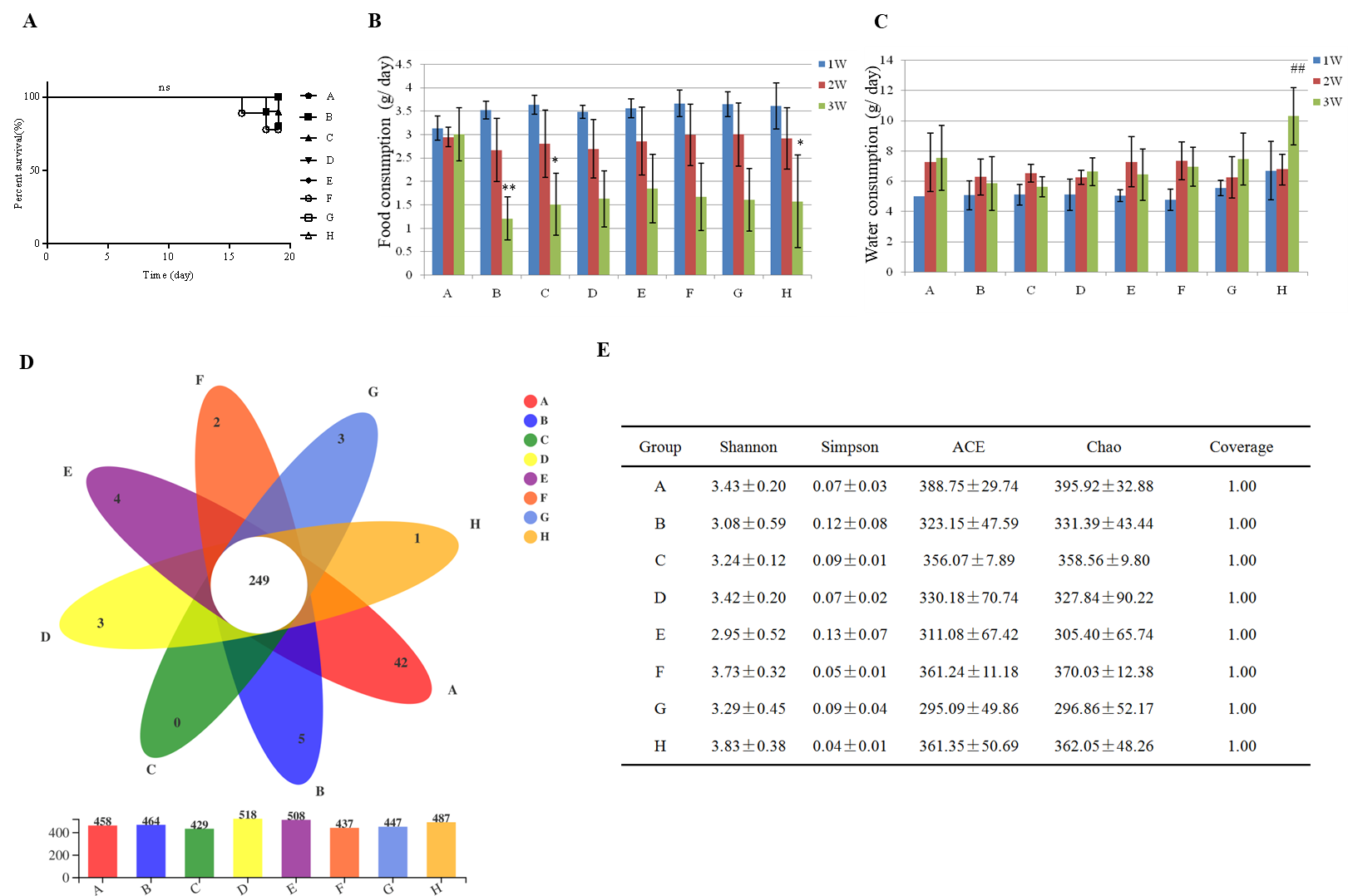


**Supplement Figure 1.** (A) The survival rate for all groups of mice. (B) Food consumption for all groups of mice. (C) Water consumption for all groups of mice. (D) Venn diagrams that illustrated observed overlap of OTUs. (E) α diversity for all groups of mice. All data are presented as the mean ± SD. *P* value <0.05 was considered to indicate statistical significance (**P* < 0.05 and ***P* < 0.01 compared with the group A, ##*P*< 0.01 compared with the group B).

**Supplement Figure 2.** Effect of SFN on *Akkermansia muciniphila* at the species level in colitis mice. All data are presented as the mean ± SD.


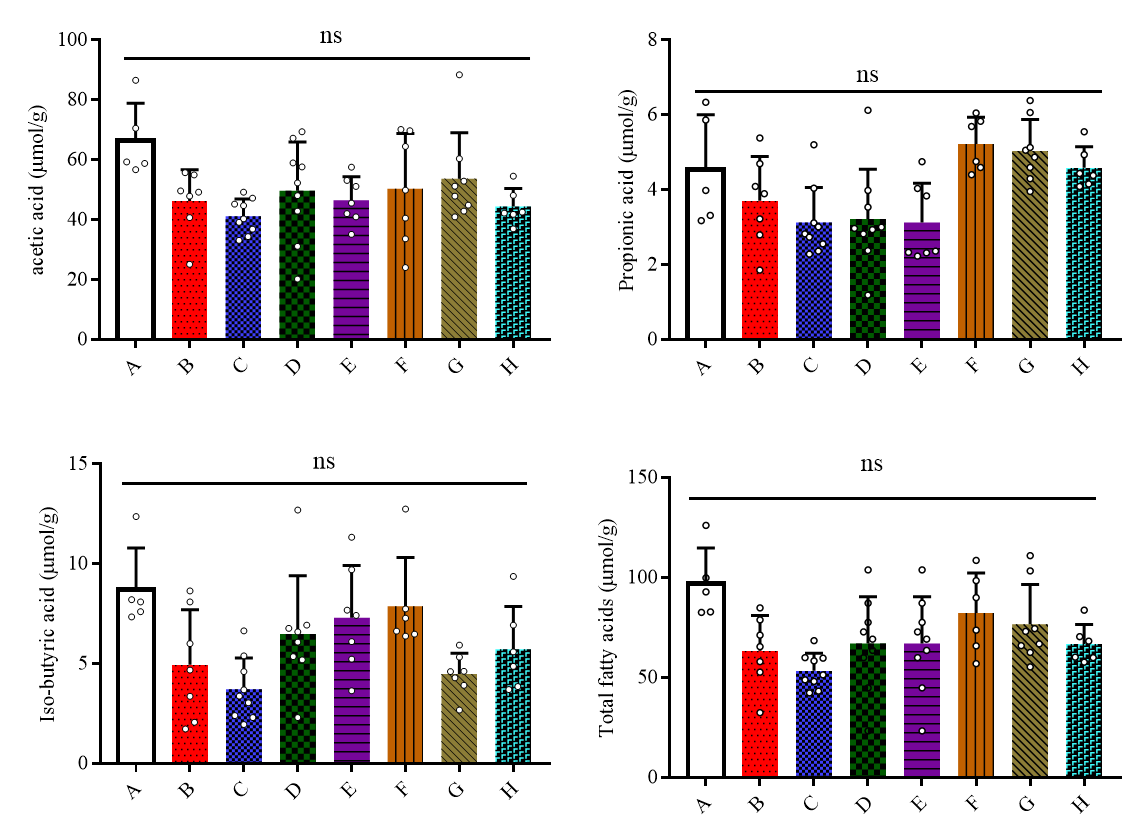


**Supplement Figure 3.** Effect of SFN on volatile fatty acids (Acetic acid, Propionic acid, Iso-butyric acid, Total fatty acids) concentrations in colitis mice. The samples were collected from colonic contents of all groups of mice. All data are presented as the mean ± SD.
